# The development and assessment of a movement-based coaching program on executive function skills: an exploratory quality improvement study

**DOI:** 10.3389/fpsyg.2026.1761762

**Published:** 2026-03-30

**Authors:** Natalie C. Wilcox, Guillermo A. Bautista, Madison A. Webb, Andre K. Hoyle, Doriana Taccardi, Hailey G. M. Gowdy, Emiko E. Patterson, Samuel E. Fredricksmeyer, Tyler G. Slade, Jaqueline R. Silva, Jim M. Carlson, Brandon P. Slade

**Affiliations:** 1 Untapped Learning Inc., Broomfield, CO, United States; 2 Pain Chronobiology and Neuroimmunology Laboratory, Department of Biomedical and Molecular Sciences, Queen’s University, Kingston, ON, Canada; 3 Sinclair Cancer Research Institute, Queen’s University, Kingston, ON, Canada

**Keywords:** adolescence, coaching, executive function, executive function disorders, mentorship, movement, physical activity

## Abstract

**Introduction:**

Executive function (EF) skills are integral cognitive processes with strong links to self-regulation and academic success. The post-COVID-19 period has seen an increase in self-reported and diagnosed EF deficits (EFDs). This quality improvement study evaluated the Untapped Learning (UL) program, a personalized, movement and mentorship-based EF program for students aged 11–24, who predominantly have self-reported EFDs.

**Methods:**

Students were paired with trained coaches and participated in weekly one-on-one sessions for the duration of one school semester. Sessions included movement and targeted EF skills training. Students completed pre-post surveys assessing five EF skills (organization, planning, communication, task completion, and mentality) across three school semesters in total.

**Results:**

Students enrolled in the program showed significant overall improvements across two out of three semesters in a younger population (11–18 years old, *n* = 222) (*p* < 0.02 Spring and Fall 2023) and in all semesters in a post-secondary (PS) age population (18–24 years old, *n* = 90) (*p* < 0.02 for all semesters). When stratified by sex, males demonstrated greater overall reported improvements than females in both cohorts. Females showed a consistent increase in planning skills in the post-secondary cohorts, indicating potential sex-specific response patterns or survey sensitivity issues.

**Conclusion:**

These findings suggest that movement-based EF coaching programs are associated with self-reported EF skill increases and highlight the need for greater sex-specificity in designing personalized mentorship programs.

## 1 Introduction

Executive function (EF) refers to the higher-order cognitive processes that are necessary to carry out complex tasks such as planning and prioritization (Diamond, 2013). Those with executive function deficits (EFDs) often struggle with self-regulation, self-control, and planning ahead (Silverstein et al., 2020). EFD is not a stand-alone diagnosis for either the International Classification of Diseases 11th Revision (ICD-11) or for the Diagnostic and Statistical Manual of Mental Disorders, Fifth Edition (DSM-5). The ICD-11 includes a diagnostic code for impaired executive functions (MB21.7), an “impairment in higher-level cognitive abilities, such as planning, sequencing, concept formation, abstracting, and decision-making”(World Health Organization, 2019). While the DSM-5 does not include a specific diagnosis and instead considers EFDs as a core feature of other diagnoses like attention deficit and hyperactivity disorder (ADHD), Autism Spectrum Disorder (ASD), anxiety, dyslexia, or other learning disabilities (Craig et al., 2016; American Psychiatric Association, 2022).

EF is a cognitive process that is modified by variations in neural structure or function (Elliott, 2003; Diamond, 2013; Craig et al., 2016; Silverstein et al., 2020; Ugarte et al., 2023). A multitude of conditions occurring at all ages may result in EFDs (Diamond, 2013). Neural injury or degeneration, learning disabilities, and mental illness have all been known to result from or be related to EFDs (Elliott, 2003). Although there is variation in the neurobiological underpinnings within the umbrella of EFDs, their core tenets: working memory deficits, inhibition, difficulties shifting between tasks, and verbal fluency (Diamond, 2013). Many assessments have been developed to measure cognitive EF abilities that are utilized for the diagnosis of EFDs (Conners et al., 1997; Gioia et al., 2000; Kamradt et al., 2019; American Psychiatric Association, 2022). Cognitive deficits in EF impact skills relevant to everyday life (Weiner et al., 2012; Kamradt et al., 2019); these EF skills are the primary focus of this study.

From a neurodevelopmental standpoint, the late childhood to adolescent period yields a high degree of brain maturation. The prefrontal cortex is responsible for utilities associated with EF and is the last part of the brain to fully mature (Arain et al., 2013). Neural maturation is mediated through a process known as ‘synaptic pruning’ in which unnecessary cortical connections within the central nervous system (CNS) are removed by the brain’s resident immune cells (Schafer et al., 2012; Wu et al., 2015). During adolescence, some areas of the brain can lose up to 50% of their synaptic connections (Rakic et al., 1994), leading to greater efficiency in information conduction and processing (Spear, 2013). On the other hand, poor synaptic pruning can lead to consequences in the efficiency of cognitive processing. For instance, variations in the synaptic pruning process have been shown to produce EFD symptoms, typical of ASD (Tang et al., 2014; Kim et al., 2017) and ADHD (Ugarte et al., 2023).

ADHD diagnosis rates increased from 6 to 10% of the American youth population between 1997 and 2018 (CDC, 2018). This number continued to jump such that in 2023 13.6% all 12–17 year olds (U.S. Census Bureau, 2023) and 14.5% of boys (Reuben and Elgaddal, 2024) (in 2022) had an ADHD diagnosis. ASD diagnoses have also skyrocketed from 0.66 to 3.23% of 8 year olds between the years 2000 and 2022 (Shaw et al., 2025). This sharp upturn in diagnoses has been attributed to multiple factors, including public awareness of the conditions (Abdelnour et al., 2022), less stringent diagnostic criteria, and the effects of the COVID-19 pandemic (Davoody et al., 2022; Auro et al., 2024). ADHD and ASD were previously thought of as childhood disorders; however, the last 20 years have shown a marked increase in adult diagnosis (Abdelnour et al., 2022; Grosvenor et al., 2024). One study found that up to 75% of people with an adult diagnosis of ADHD did not have a diagnosis as a child (Faraone et al., 2004). Not only does this indicate an increase in the diagnostic rate of EFDs, but it also shifts the typical demographic to an older subset. Considering that many people do not ‘outgrow’ their childhood or adolescent EFDs, there is an increasing need for EF supports that not only benefit children but also their transition to adulthood.

### 1.1 Literature review: improving EF skills

Many EFDs are traced to a deficit in catecholamine neurotransmitter regulation (Hosenbocus and Chahal, 2012), which has been related to variations in the brain’s reward pathways (Ventura et al., 2007). People with EFDs often have a decreased amount of dopamine in particular, which makes the initiation of tasks more difficult (Volkow et al., 2011). The supplementation of dopamine through stimulant drugs has been beneficial to many, in particular those with ADHD, and has been reported to increase focus depth and duration (Faraone and Biederman, 2002). Despite the physiological underpinnings of the disorder it has been reported that EF skills can be improved through targeted therapeutics such as games and activities (Blair, 2017).

Aerobic exercise has consistently been shown to improve EF functionality in children and adolescents (Best, 2010). This improvement is reported for both acute and chronic exercise interventions; with the greatest improvements seen when physical and cognitive tasks were integrated (Best, 2010). For example, in one study young participants who rode an exercise bike while watching an age appropriate television show reported increases in all EF measures in the exercise cohort when compared to the control that watched the show without exercising (Ellemberg and St-Louis-Deschênes, 2010). Similarly, it has been shown that memory recall improvement is generated from team game-based exercise rather than aerobic exercise alone (Pesce et al., 2009). Thus, this evidence suggests that physical activity may aid in the development of sustained attention mechanisms (Best, 2010). Infants who had less movement-attention integration at 1–3 months of age were more likely to have attention problems at 8 years old (Friedman et al., 2005). These early development studies suggest that the combination of movement and EF is innate and may explain the benefits that are seen when physical and cognitive tasks are integrated.

At its conception, the UL EF program was conducted solely in-person and integrated ‘movement breaks’ throughout the session which could consist of a walk outside, a game of ping pong, or throwing a football. For students who access the program in-person, which accounts for most of the 6th–12th grade (6–12) cohort, this remains a key part of the UL program. However, 90% of post-secondary (PS) students access UL resources remotely due to attendance at PS institutes across North America. Movement remains a key component of the PS program, but is accessed in different modalities than the in-person students. Many coaches help students schedule movement time into their days to not only receive the EF benefits of physical activity during the session, but also throughout their week.

### 1.2 Sex differences in EFs

The prevalence of female EFD diagnosis and involvement in EFD research is markedly lower than in the male population. Recent research has begun to acknowledge the presence of females with ADHD who often present differently than their male counterparts (Skogli et al., 2013; Tetering et al., 2020). Females with ADHD are less likely to express hyperactivity, rather they often display a greater inattentive phenotype. On average females tend to have higher EF skills which makes deficits from the norm less apparent to parents and teachers, leading to a lack of diagnosis in this population (Hinshaw et al., 2022). Female adolescents overall tend to have a higher degree of self-regulation, which is evidenced by less involvement in gambling, fatal accidents, and crime (Tetering et al., 2020). Adolescent females between the ages of 13–15 years old are also more likely to self-report higher self-regulation capacity than their male counterparts (Tetering et al., 2020). Females are also less likely to be involved in EFD-specific interventions, particularly those that are physical activity-based (Slobodin and Davidovitch, 2019). A recent review article investigated the prevalence of physical exercise among children and adolescents with ADHD or ASD. Of note, out of the 17 studies included only 11 included girls, and from those, only three studies had ≥50% females (Grahn, 2025). This highlights the need for female inclusion in EF interventions and studies.

### 1.3 The impact of the COVID-19 pandemic on EF

The COVID-19 lockdown in the United States forced students to abandon their regular school lives for a virtual academic system. For many, this decreased the availability of peer-to-peer interactions while increasing accessibility to distractions such as video games and social media. Extracurricular activities such as sports were also disrupted or canceled during lockdowns, exacerbating the decline in childhood physical activity trends (Guthold et al., 2020). Physical activity is an integral piece of neural development and has been shown to improve focus in children with ADHD and EFD (Li et al., 2023). The emergence of online learning coupled with a decrease in physical activity may have contributed to the increased rate of EFDs seen in adolescents during the COVID-19 pandemic (Chichinina and Gavrilova, 2022). During this difficult time, college and high school students have been experiencing difficulties in transitioning to independence (Thompson et al., 2021), completing academic work (Worsley et al., 2021), advocating for themselves (Pfeifer et al., 2021), communicating with peers (Worley et al., 2023), managing large projects with multiple sub-tasks, balancing daily life responsibilities, and maintaining a positive mindset (Usán et al., 2022). These challenges not only have affected academic performance but also have impacted students’ personal growth and future career prospects.

### 1.4 EF training and mentorship

Peer mentorship is one of the core tenets of the UL program. The mentorship experience is dependent on the student-coach pairing and will be unique to the student’s situation. Many students join the program with high proficiency in some EF skill areas (Slade, 2024c), and the coach will primarily focus on the EF skills that the student struggles most with. Although all mentors receive the same training each student’s experience will be individual to them, focusing on interpersonal connections with their mentor and tailored to the EF areas they need to improve most (Slade, 2024c).

Failing one or more classes can be a catalyst for students to join UL (Slade, 2024a). Following a class failure, students often develop a negative attitude toward themselves (Filozof et al., 1998). When a student does not believe that they can succeed, even the best resources may not help their growth (Gao and Ali, 2024). This is why UL aims to support the student as a whole. Coaches take time getting to know students’ interests and personalities, to encourage and support them in a way that will help them to gain academic skills but also regain their belief in their own abilities (Untapped Learning, 2025). Coaches practice hard conversations with students, so that rather than backing away from these situations, students know that they have the skills to tackle them. They also work on managing stress by taking breaks and participating in breathing exercises rather than allowing stress to halt all forward progress. Coaches are also in the unique position of being a compassionate peer rather than a teacher or parent. Oftentimes, just hearing that they can succeed from an external source is a benefit to the students’ mentality (Lyons and Yi, 2025).

### 1.5 Present study

The UL program integrates both physical activity and targeted EF coaching (Slade, 2024b; Untapped Learning, 2025). The decision to collect data on the program stems from the commitment to continuous program improvement. Through analysis and interpretation of the findings, this study aims to advance the field’s understanding of effective strategies for improving EF skills in adolescents and young adults. This study aims to answer whether participation in the UL program can improve self-reported EF skills that apply to daily life. Secondarily, we aim to understand whether demographic factors such as age group and sex are associated with differential patterns of change. We hypothesized that participants would report an increase in overall EF skills following program participation, with potentially greater increases in males, who represent the primary client base of the program.

## 2 Methods

### 2.1 Participants

**Figure 1:**
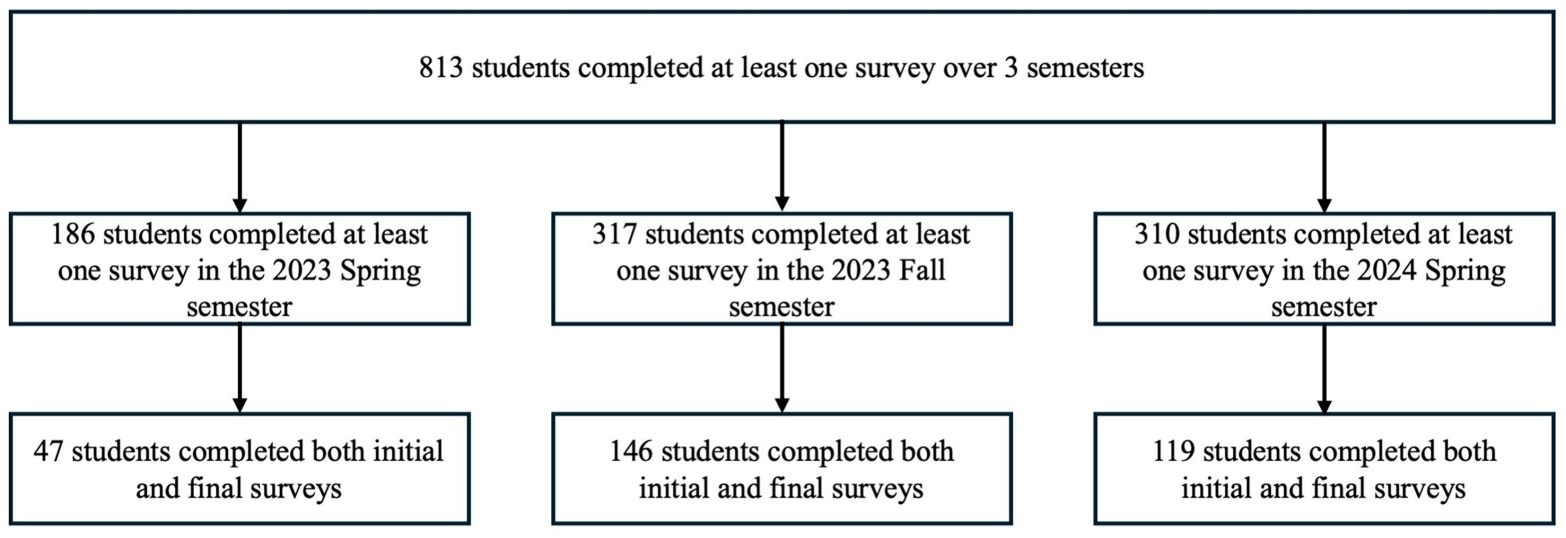
Flow chart of participant inclusion. Student attrition throughout each semester of assessment.

The survey was administered to consenting registered participants of the UL program between the ages of 11 and 24 years, the majority of whom were enrolled in school grades 6–12, or PS education. Within each semester, between 68.91–73.29% of the population was enrolled in the 6–12 program (Table 1). Both the 6–12 and PS programs had a larger population of male students each semester (6–12: 67.29–81.71%, PS: 59.46–85.71%) (Table 1). Sex designation refers to the self-reported marker at the time of enrollment in the program. Participation was completely voluntary. Students in the UL program were enrolled for a semester at a time. Therefore, there were different cohorts of students over the three semesters: Spring 2023 (S23), Fall 2023 (F23), and Spring 2024 (S24) (Figure 1).

**Table 1:** Demographic table.

	Age group	Sex	N	% of reporting sample
Spring 2023				
	6th–12th grade			
		Male	25	78.13%
		Female	8	21.88%
	Post-secondary			
		Male	12	85.71%
		Female	2	14.29%
Fall 2023				
	6th–12th grade			
		Male	72	67.29%
		Female	35	32.71%
	Post-secondary			
		Male	24	61.54%
		Female	15	38.46%
Spring 2024				
	6th–12th grade			
		Male	67	81.71%
		Female	15	18.29%
	Post-secondary			
		Male	22	59.46%
		Female	15	40.54%

Grade level and sex data were recorded from initial program registration.

#### 2.1.1 Attrition analysis

Within each semester, 36.15–63.76% of students who completed an initial survey also completed a final survey (Supplementary Figure 2). Among the students who completed an initial survey 2.31–9.02% of students left the program before completing a final survey; the remaining 30.57–61.5% of students remained in the program, but did not complete a final survey. Survey completion rates varied greatly between semesters, with the highest completion rates in the F23 semester. The declining completion rates in the spring semesters likely reflects increased academic demands when post-surveys coincided with final examination preparations and project deadlines. To assess potential selection bias, Fisher’s exact tests with 3×2 contingency tables were utilized to compare the demographics and completion patterns within each semester.

##### 2.1.1.1 Completion: sex differences

Sex patterns varied across semesters (Supplementary Table 1). The S23 and F23 semesters had no statistically significant sex differences, however the S23 semester had higher male completion rates, whereas the F23 semester had higher female completion. The S24 semester showed a statistically significant sex difference, with males demonstrating higher completion (55.28% vs. 36.14% for females).

##### 2.1.1.2 Completion: age group differences

Age group patterns were more consistent than sex patterns. PS students demonstrated non-significantly higher completion rates in all three semesters. The pattern was primarily driven by 6–12 student non-completion rather than program attrition.

Initial survey baseline EF scores were not significantly different by completion group or semester (Supplementary Figure 2).

### 2.2 The Untapped Learning program

#### 2.2.1 Mentorship

When a student joins the program, they are paired with a coach following an interview with a senior manager. The interview aims to identify difficulties, motivations, and interests for each student. They are then assigned to a coach who is the same gender as them (whenever possible) and has similar interests. The coaches are typically between 3 and 10 years older than the students as it has been shown that students respond well to peer-mentorship, are more likely to bond with the coach, and will be less resistant to the intervention (Graham et al., 2022; Le et al., 2024). Students in the 6–12 program are typically paired with current university students, while PS students are paired with university graduates. All coaches undergo extensive training provided by UL.

The coach training was developed by senior UL managers and consists of online modules within the categories of understanding EF, mentee/coach relationships, parent communication, and the UL process. There are topics including how to help a student plan for their week, how to navigate academic accommodations, communicating with parents, resources on coach-student relationships and mandatory reporting, among others. Each section has a quiz, a video, a PowerPoint and a written text so that coaches can access the information in a way that works best for them. Each coach must pass each quiz before being allowed to work with students. Coaches are advised to go through the content on their own prior to the company-wide in-person training. Prior to the new school year, a coach training event is held to train new coaches and introduce new policies. At this event, senior managers will discuss how to handle many situations, from parent interactions to helping students work on their EF skills outside of the school environment. For the remainder of their time with UL coaches will have weekly meetings with their manager who will provide additional training and support. The training overall was aimed to prepare coaches to assist in the development of a variety of EF skills, which were the same as those assessed by the biannual survey. Upon joining the program students identify the key skills they wish to work on, however, as time progresses and their skills improve the focus areas may shift.

#### 2.2.2 Training EF skills

##### 2.2.2.1 Organization

The category of organization includes physical and digital items as well as thoughts. Coaches start their relationship with students by having them create physical organization systems for papers they receive in class and a folder system for their digital files (Table 2). Most students access and complete their assignments online; therefore, it is necessary for students to be able to organize their electronic files. Coaches will help students to create and optimize a system that works best for their academic and personal needs (Table 2). Throughout the mentoring relationship, the coach will check in with the student and often give them time to re-adjust their systems until the student is able to maintain their organization independently.

**Table 2:** Example interventions used by UL coaches to target specific EF skills.

EF skill	Sub-skill	Intervention
Organization	Physical organization	Creating a HW folder “to turn in” and “turned in” to help students physically organize their assignments, reducing the likelihood of lost or forgotten work.
	Electronic organization	During sessions where a student has no work, focus on organizing their digital lives. This includes cleaning up their email inbox, and notifications, and organizing Google Drive.
	Organizing thoughts	Guide students in effectively using physical or digital planners to track assignments and deadlines.
	Workspace organization	Help students set up and maintain an organized study area at home
Planning	Weekly planning	Coaches guide students through planning their week, which includes tasks to reach their academic and personal goals.
	Breaking down big projects	Teach students to “chunk” larger projects into smaller, manageable tasks. This not only helps with planning but also reduces the anxiety that comes with large assignments.
	Backwards planning	For students interested in goals outside academics, such as starting a YouTube channel or a fitness routine, coaches help them create a backward plan to map out the steps needed to achieve these goals.
	Time-blocking	Teach students to allocate specific time blocks for different tasks or subjects
Communication	Advocacy	Practicing how to talk to teachers or professors through role-play helps students develop the confidence to communicate their needs effectively.
	Respectful emailing	During sessions, coaches can guide students on how to write clear and effective emails, especially when communicating with teachers/professors about assignments, accommodations, or other academic concerns.
Completion	To-do lists	Coaches will help students to make a to-do list, so that when they finish a task or assignment, they can check it off the list. This way the student can ensure that they have completed everything that they needed to do.
	Time blocking	It is common for completion to become an issue for students when they feel they do not have enough time to finish everything that they need to do. Coaches help students to find areas in their daily schedule to designate to particular tasks. This way the student only needs to follow the plan in order to complete everything.
	Creating work routines	Sometimes even when students have a designated time to complete a task it will not get completed because they get distracted while working. By working with the mentor, a student can establish a working routine to set themselves up to be productive and not get distracted while working. This often involves sitting in the same place and removing distracting technology.
Mentality	Growth Mindset	In every session, particularly in challenging conversations, coaches emphasize a growth mindset. They focus on praising the student’s effort and process rather than just outcomes, helping to build resilience and a positive attitude toward learning.
	Practicing hard conversations	Coaches use role-playing to help students prepare for challenging situations, such as advocating for themselves or managing their time. This builds confidence and helps students approach these situations with a positive and proactive mindset.
	Stress management	Teach breathing exercises, mindfulness, or other stress-reduction strategies

Example interventions that may be utilized by an UL coach for targeting specific skills. Coaches may use other techniques, however this represents the main teachings that the program utilizes.

##### 2.2.2.2 Planning

The skill of planning also interacts with the previous skill of organization. Considering that many students with ADHD or EFDs miss assignment deadlines and suffer from lower grades than their peers (Jangmo et al., 2019), coaches frequently examine academic assignments for the week with their student and help them create a weekly plan (Table 2). This plan includes everything they need to get done for the day, when they will do each assignment, and when their classes are. For larger assignments coaches also help students convert assignments into shorter sections to create multiple mini-deadlines. For example, if the student was assigned a 12-page research paper, the coach may help the student make a deadline for finding the papers for the review, a deadline for the outline, a separate deadline for the introduction paragraph, and as many more deadlines as needed to break the assignment down from planning to final edits (Table 2). Learning the planning process often begins with coaches teaching the planning methods and evolves into the students planning independently with coach support.

##### 2.2.2.3 Communication

Students with EFDs often receive academic accommodations; however, many universities require students to know and request adherence to their individual accommodations (Pfeifer et al., 2021). This is a key element of the communication EF skill (Table 2). Students need to know how to ask for what they need respectfully and clearly. Coaches and mentees identify a potential future scenario, such as emailing a professor to ask for an extension on an assignment per their accommodations. The coach may provide the student with a template email and have the student practice writing what they would say. Communication skills are often strengthened as other EF skills develop because working memory, planning and organization are all needed for strong advocacy (Nilsen and Bacso, 2017). Over time, the coach and student will repeat this role play in alternative scenarios until the student is comfortable advocating alone.

##### 2.2.2.4 Completion

People with EFDs often struggle with procrastination, especially in post-secondary institutions where deadlines are often more strictly upheld (Santelli et al., 2020). To support students’ timely assignment completion, coaches help to create frequent to-do lists. Many students have reported that having a to-do list decreases their academic stress, because they can “have the paper hold all the things that need to be done, rather than holding it all in [their] brain”- post-secondary student, age 21. Students with EFD often struggle with working memory (Diamond, 2013), which causes greater difficulties recalling tasks. In addition to the to-do list, coaches teach students how to go through their weekly calendar and strategize times to complete work. Over time students learn to gauge how long it takes them to complete a task and can learn how to utilize their schedule effectively.

##### 2.2.2.5 Mentality

At UL, mentality is used as a distinct construct, measuring student affective well-being, metacognitive awareness, and growth-mindset (Dweck, 2006). Unlike organization, planning, communication and completion, mentality is not a well-defined EF process (Diamond, 2013). However, it remains an integral part of the UL program. Its inclusion in the composite EF score reflects its programmatic importance rather than its adherence to theoretical EF frameworks. In the UL model, motivational and affective factors, which are measured by the “mentality” questions, are often facilitators of overall EF growth as measured by the other sub-skills. Previous research demonstrates that affective and metacognitive awareness and self-control are important for bridging the gap between EF and academic interventions and real-world performance (Zelazo and Carlson, 2012; Duckworth et al., 2019). Therefore, we acknowledge that although mentality does not constitute a true EF skill, it continues to play an integral role in the enablement of EF skill development.

At UL peer mentorship plays an important role in the development of mentality. Coaches are trained to not only praise overt successes, but also their work toward a goal regardless of outcome (Table 2). Mentality also covers stress management, by using stress as a tool but not as an overwhelming state. Coaches can teach students breathing exercises, set aside time in sessions for metacognitive reflection, or integrate physical activity or art as other stress reduction techniques, as needed by the student (Table 2). As with the other EF skills the goal is for students to gradually use these techniques independently from their mentor’s prompting.

### 2.3 Study design and data collection

As a quality assurance and improvement study, the collected data was used to modify coach training and company practices. The primary goal of data collection was to improve the program internally. Therefore, the survey was created in-house to assess EF growth in the UL cohorts. Although considered, validated EF measures such as the Behavior Rating Inventory of Executive Function (BRIEF) or Barkley Deficits in Executive Functioning Scale (BDEFS) were not utilized for several reasons. These resources require licensed administration, specialized training, and per-use fees, which were not feasible given the program’s operational scale and resource constraints. Additionally, the BRIEF relies on parent/teacher informants (Gioia et al., 2000; Gioia et al., 2015), which was not feasible for an internal program assessment. The BDEFS is normed to an adult population (Barkley, 2011), although a version exists for children and adolescents (BDEFS-CA) (Barkley, 2012), administering two separate measures was not practical given the operational constraints.

Internal consistency of the survey used was assessed using Cronbach’s *α* for each EF subskill across the pooled sample (Supplementary Table 2). Reliability was variable, with all-question values ranging from 0.14 (mentality) to 0.64 (organization). Further exploratory analysis revealed that low reliability was primarily driven by reverse-coded questions. Excluding these questions in a subsequent Cronbach’s α assessment improved scores to a range of 0.57–0.88. Organization (*α* = 0.82), planning (*α* = 0.73), and communication (*α* = 0.88) demonstrated acceptable to good internal consistency, while completion (*α* = 0.57) and mentality (*α* = 0.60) remained borderline. This suggests that completion and mentality are less coherently captured by the created survey. All reported analyses for the paper were conducted on the entire question set, including reverse-coded questions, to maintain analytical consistency. As the survey was developed as an internal program-monitoring tool and not as a psychometric instrument, subskill findings should be interpreted with an appropriate level of caution.

The survey was administered twice a semester as an “initial” and “final” assessment to measure the students’ EF growth while in the program. It was conducted over three semesters, S23, F23, and S24. The survey was originally designed to improve the quality of the UL program offering. The methods of completion were: (1) pen and paper format: students were asked to circle their answers along a 1–5 Likert scale; (2) digital, using a survey link through an app (Boost Accountability Inc.) or using a Google Form questionnaire. The questions asked remained consistent across all timepoints and access methods. The survey was divided into 5 sections: organization, planning, completion, communication, and mentality. Each section had 5 questions with a potential score from 1 to 5. Each section was scored by averaging the responses from each question. All question scores were adjusted *post hoc* such that a higher score indicated greater mastery of the given skill. The average of all sections’ scores was used as an overall EF Score. The maximum score was a 5 and the minimum was a 1 for each section and overall EF score.

Coaches administered the initial survey between the first 2 weeks of the semester and the final survey in the 3 weeks prior to final exam administration at the majority of schools. All coaches were required to attend a teaching session hosted by the UL managers on the reasoning for surveys and proper administration conduct. All surveys were to be administered at the beginning of sessions prior to starting the coaching for the day. To ensure confidentiality, coaches would exit the room while students completed the survey and would re-enter after the student indicated that they were finished. Completion times were 5–15 min on average.

### 2.4 Data analysis

To determine if students self-reported higher EF skills after participating in the program, matched t-tests were used to compare overall EF scores within each semester. Further stratification was done to understand which EF skill categories were increased by the program overall. We separated further by self-reported sex and program enrollment. Paired t-tests were performed to assess any changes from initial to final timepoints. Comparisons were conducted to understand differences between male and female cohorts (PS and 6–12 male and female) in the initial and final surveys among semesters using a one-way ANOVA with Tukey’s multiple comparisons test. The two age cohorts were compared at both the initial and final timepoints with semesters pooled using an unpaired t-test. To understand how subskill growth was correlated with one another, the change in EF category scores was recorded for each student within either the PS or 6–12 cohort, combining all semesters and both sexes. A non-parametric Spearman correlation was conducted and Spearman’s r values and corresponding *p* values were collected. Attrition analysis utilized a one-way ANOVA with a Tukey’s multiple comparison test to assess differences in EF baseline scores. Categorical comparisons, such as attrition rates by sex and age, were compared using a Fisher’s exact test. A significant value was considered when the *p*-value was less than 0.05. All analysis was conducted using Prism version 10.

### 2.5 Ethics approval

Due to the nature of Quality Assurance/Improvement, the study was initially exempted from Research Ethics Board Review. It was approved by Queen’s University Health Sciences and Affiliated Teaching Hospitals Research Ethics Board for secondary use of data for the purpose of this paper (File number: 6041922) on August 19th, 2024 (Renewed August 19th, 2025).

## 3 Results

### 3.1 Pre-post analysis

**Figure 2:**
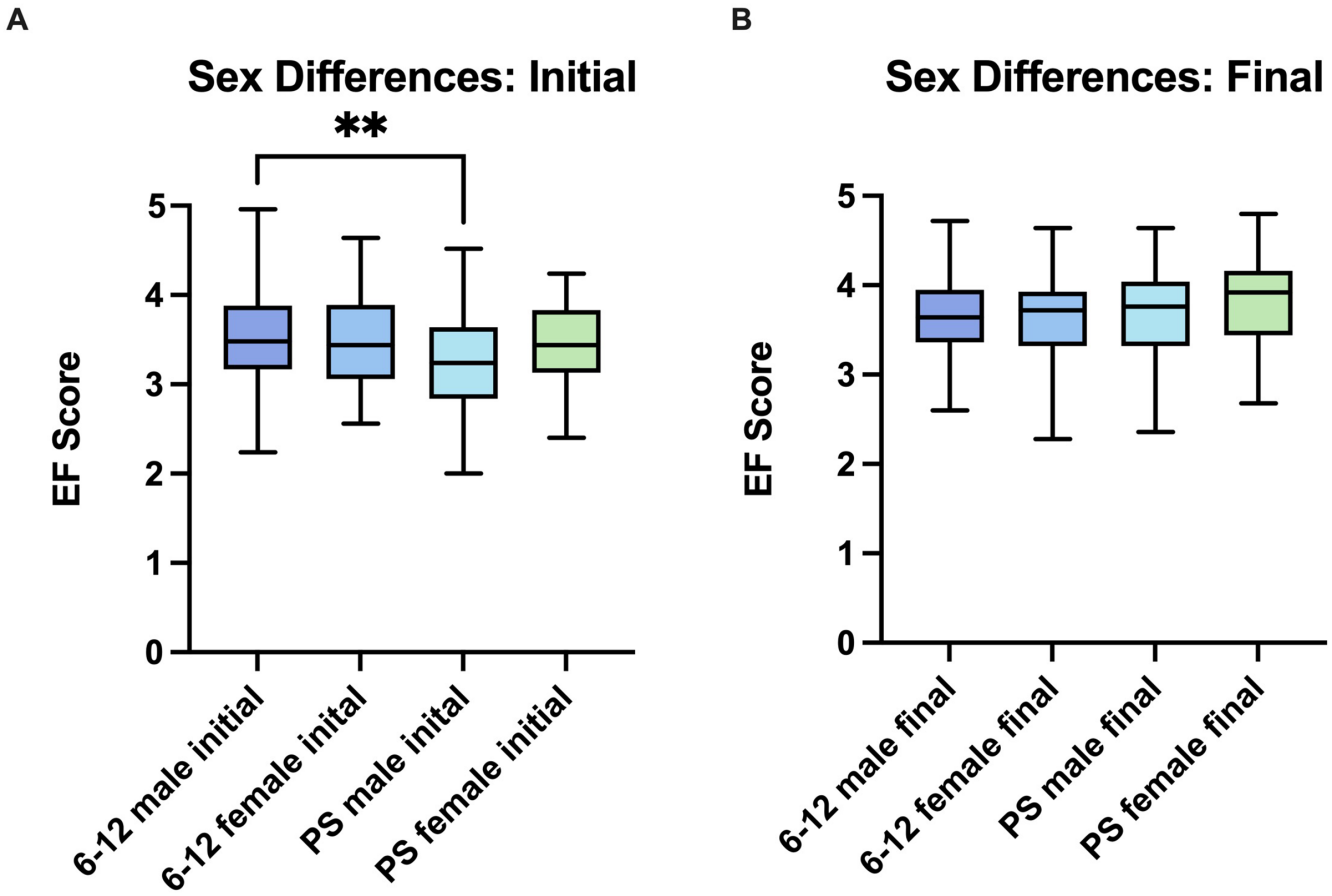
Initial and final scores are consistent between the sexes in each age group. **(A)** Comparison of initial overall EF scores between sexes and age groups using one-way ANOVA with Tukey’s multiple comparisons test. 6–12 males initial (*n* = 164) *M* = 3.52 (0.50), 6–12 females initial (*n* = 58) *M* = 3.46 (0.50), PS males initial (*n* = 58) *M* = 3.23 (0.58), PS females initial (*n* = 32) *M* = 3.44 (0.47). **p < 0.01. **(B)** Comparison of final overall EF scores between sexes and age groups using one-way ANOVA with Tukey’s multiple comparisons test. 6–12 males final (*n* = 164) *M* = 3.65 (0.43), 6–12 females final (*n* = 58) *M* = 3.64 (0.48), PS males final (*n* = 58) *M* = 3.72 (0.48), PS females final (*n* = 32) *M* = 3.84 (0.52).

Both 6–12 and PS cohorts showed significant increases in reported EF skills after one semester, with the exception of the S24 semester for the 6–12 cohort (Table 3). When stratified by sex, males in the S23 and F23 semesters consistently reported a statistically significant improvement in EF scores. In F23 PS, females also had a significant increase in EF skill growth (Table 3). All groups showed an upward trend with no decreases in overall EF skill measurements in any given semester. Initial scores ranged from 3.11 (± 0.54) to 3.76 (± 0.06) with PS males 3.23 (± 0.58) scoring significantly lower than 6–12 males 3.52 (± 0.50, *p* = 0.0012). Final scores converged to a narrowed range between cohorts (3.65 ± 0.43 to 3.81 ± 0.52) with no significant differences between groups (Figure 2B).

**Table 3:** The UL program improves overall EF skills in both the 6–12 and PS COHORTS.

Group	Spring 2023						Fall 2023						Spring 2024					
	Initial		Final		*p*	*d*	Initial		Final		*p*	*d*	Initial		Final		*p*	*d*
	M	SD	M	SD			M	SD	M	SD			M	SD	M	SD		
6–12 overall	3.36	0.34	3.53	0.40	**0.0033**	0.53	3.47	0.51	3.67	0.48	**<0.0001**	0.44	3.59	0.53	3.67	0.40	0.1045	0.18
6–12 female	3.45	0.23	3.62	0.42	0.2039	0.5	3.41	0.53	3.61	0.55	**0.0300**	0.38	3.61	0.50	3.72	0.35	0.2658	0.3
6–12 male	3.34	0.36	3.51	0.40	**0.0098**	0.54	3.52	0.47	3.70	0.45	**0.0003**	0.44	3.59	0.54	3.64	0.42	0.4211	0.1
PS overall	3.30	0.45	3.87	0.40	**0.0023**	1.01	3.21	0.54	3.77	0.52	**<0.0001**	0.84	3.40	0.56	3.72	0.51	**0.0099**	0.45
PS female	3.76	0.06	4.08	0.11	0.0792	5.66	3.38	0.49	3.92	0.61	**<0.0001**	1.41	3.46	0.45	3.73	0.44	0.1116	0.44
PS male	3.22	0.44	3.83	0.42	**0.0048**	1.02	3.11	0.54	3.68	0.45	**0.0004**	0.83	3.36	0.62	3.71	0.56	**0.0470**	0.45

For assessing EF growth within each semester paired *t*-tests were conducted on the mean of all EF skill scores (EF score). Bolded text indicates *p* < 0.05.

Subskill analysis revealed an increase in almost every skill in the PS cohort but inconsistent patterns in the 6–12 cohort (Tables 4, 5). Overall, in the 6–12 cohort, there was inconsistent skill improvement, which varied by semester with no uniform pattern. Stratification by sex showed females improving in mentality (S23), whereas males improved in mentality (S23 and S24), organization (F23), completion (F23), and communication (F23) (Table 4).

**Table 4:** EF skill growth was sporadic in the 6–12 cohort when stratified by semester and skill.

Group	EF skill	Spring 2023						Fall 2023						Spring 2024					
		Initial		Final		*p*	*d*	Initial		Final		*p*	*d*	Initial		Final		*p*	*d*
		M	SD	M	SD			M	SD	M	SD			M	SD	M	SD		
6–12 overall																			
	Organization	3.79	0.44	3.86	0.62	0.4071	0.15	3.7	0.65	3.88	0.65	**0.0023**	0.30	3.8	0.67	3.87	0.58	0.2463	0.13
	Planning	3.05	0.67	3.14	0.69	0.4017	0.15	3.15	0.72	3.38	0.74	**0.0044**	0.28	3.24	0.78	3.41	0.62	0.0755	0.20
	Completion	3.31	0.34	3.11	0.47	0.0614	−0.33	3.09	0.58	3.28	0.72	**0.0438**	0.24	3.23	0.73	3.24	0.65	0.9429	0.01
	Communication	3.57	0.66	3.76	0.78	0.1091	0.29	3.71	0.75	3.90	0.78	**0.0031**	0.29	3.76	0.78	3.89	0.76	0.0621	0.21
	Mentality	3.16	0.67	3.78	0.65	**0.0005**	0.69	3.88	0.72	3.93	0.61	0.3706	0.09	3.62	0.57	4.04	0.56	**<0.0001**	0.77
6–12 female																			
	Organization	3.89	0.23	3.89	0.72	>0.9999	0.04	3.58	0.78	3.79	0.72	0.0516	0.34	3.69	0.87	3.89	0.73	0.1489	0.39
	Planning	3.46	0.5	3.46	0.85	>0.9999	0	3.14	0.82	3.48	0.74	0.0196	0.41	3.45	0.75	3.53	0.54	0.5919	0.14
	Completion	3.23	0.33	3.10	0.45	0.5368	−0.23	3.01	0.61	3.27	0.79	0.0741	0.31	3.24	0.78	3.23	0.60	0.9584	−0.01
	Communication	3.63	0.62	3.8	0.8	0.6002	0.26	3.61	0.70	3.77	0.81	0.292	0.18	3.95	0.68	4.04	0.78	0.5327	0.17
	Mentality	3.11	0.32	3.8	0.46	**0.0247**	1.25	3.69	0.6	3.72	0.62	0.7898	0.05	3.71	0.7	3.91	0.42	0.1901	0.36
6–12 male																			
	Organization	3.76	0.48	3.86	0.61	0.3273	0.2	3.76	0.58	3.92	0.61	**0.0199**	0.28	3.82	0.62	3.86	0.55	0.5538	0.07
	Planning	2.94	0.67	3.06	0.63	0.3497	0.19	3.16	0.67	3.33	0.74	0.073	0.21	3.19	0.78	3.38	0.64	0.0919	0.21
	Completion	3.33	0.34	3.12	0.49	0.0815	−0.35	3.09	0.5	3.29	0.71	**0.0345**	0.25	3.2	0.73	3.24	0.66	0.9156	0.01
	Communication	3.55	0.70	3.75	0.8	0.1288	0.31	3.76	0.77	3.97	0.76	**0.0018**	0.38	3.72	0.80	3.86	0.75	0.0808	0.22
	Mentality	3.18	0.74	3.77	0.7	**0.005**	0.62	3.97	0.76	4.03	0.59	0.3635	0.11	3.6	0.54	4.06	0.58	**<0.0001**	0.89

For assessing EF skill growth within each semester in the 6–12 cohort, paired t tests -were conducted on the mean score of all initial and final survey questions falling within each skill category. Bolded text indicates *p* < 0.05.

**Table 5:** The UL program improves EF skills in the overall, male, and some of the female PS cohort.

Group	EF skill	Spring 2023						Fall 2023						Spring 2024					
		Initial		Final		*p*	*d*	Initial		Final		*p*	*d*	Initial		Final		*p*	*d*
		M	SD	M	SD			M	SD	M	SD			M	SD	M	SD		
PS overall																			
	Organization	3.31	0.55	3.91	0.52	**0.0087**	0.82	3.43	0.61	4	0.58	**<0.0001**	0.80	3.71	0.8	4.05	0.68	**0.0227**	0.39
	Planning	3.44	0.86	4.09	0.52	**0.0116**	0.78	2.96	0.77	3.81	0.72	**<0.0001**	0.91	3.18	0.8	3.75	0.81	**0.0006**	0.62
	Completion	3.26	0.42	3.37	0.38	0.4662	0.20	2.67	0.66	3.24	0.74	**0.0010**	0.56	2.87	0.72	3.16	0.77	**0.0193**	0.40
	Communication	3.09	0.74	3.9	0.81	**0.0016**	1.06	3.37	0.79	3.90	0.69	**<0.0001**	0.74	3.47	0.73	3.69	0.79	0.1098	0.27
	Mentality	3.39	0.47	4.06	0.57	**0.0073**	0.85	3.67	0.59	3.96	0.56	**0.0107**	0.43	3.8	0.6	3.92	0.37	0.2562	0.19
PS female																			
	Organization	3.7	0.42	3.3	0.14	0.2952	1.41	3.64	0.61	4.21	0.41	**0.0005**	1.16	3.96	0.57	4.09	0.62	0.4849	0.19
	Planning	4.4	0.57	4.4	0.28	>0.9999	0.00	3.25	0.64	3.97	0.82	**0.0019**	0.98	3.37	0.74	3.88	0.72	**0.0222**	0.66
	Completion	3	0.57	3.6	0.28	0.2048	2.12	2.84	0.58	3.33	0.82	**0.0030**	0.92	2.77	0.74	3.16	0.65	0.0525	0.55
	Communication	4.2	0.85	4.7	0.14	0.4662	0.71	3.37	0.84	3.96	0.78	**0.0048**	0.87	3.43	0.67	3.68	0.73	0.2227	0.33
	Mentality	3.5	0.14	4.4	0.57	0.3228	1.27	3.8	0.48	4.12	0.67	**0.0105**	0.76	3.79	0.45	3.84	0.42	0.7387	0.09
PS male																			
	Organization	3.25	0.55	4.02	0.49	**0.0016**	1.20	3.29	0.59	3.86	0.63	**0.0027**	0.69	3.54	0.9	4.02	0.72	**0.0269**	0.51
	Planning	3.28	0.81	4.03	0.54	**0.01**	0.90	2.78	0.8	3.71	0.66	**0.0002**	0.89	3.05	0.9	3.66	0.88	**0.0104**	0.60
	Completion	3.3	0.4	3.33	0.39	0.8434	0.06	2.56	0.69	3.18	0.7	**0.0004**	0.82	3.94	0.71	3.20	0.85	0.1539	0.32
	Communication	2.9	0.56	3.77	0.8	**0.003**	1.10	3.37	0.77	3.87	0.65	**0.0037**	0.66	3.49	0.78	3.7	0.84	0.2913	0.23
	Mentality	3.37	0.5	4	0.57	**0.0224**	0.77	3.58	0.64	3.86	0.47	0.11	0.34	3.8	0.69	3.97	0.33	0.2577	0.25

For assessing EF skill growth within each semester in the 6–12 cohort, paired t tests were conducted on the mean score of all initial and final survey questions falling within each skill category. Bolded text indicates *p* < 0.05.

Upon stratification by skill in the PS cohort overall, all the subsections were significant except for completion (S23) and, communication and mentality categories in S24. When separated by sex, both the male and female groups had some significant improvement within each semester, with the exception of the S23 semester for the female cohort. The female cohort showed an increase in planning in both the F23 and the S24 semesters, but not the S23 semester (Table 5). The male cohort showed broader gains, with organization and planning improving every semester, whereas completion, communication, and mentality improved in a semester-specific manner (Table 5).

### 3.2 Sub-skill correlations

**Figure 3:**
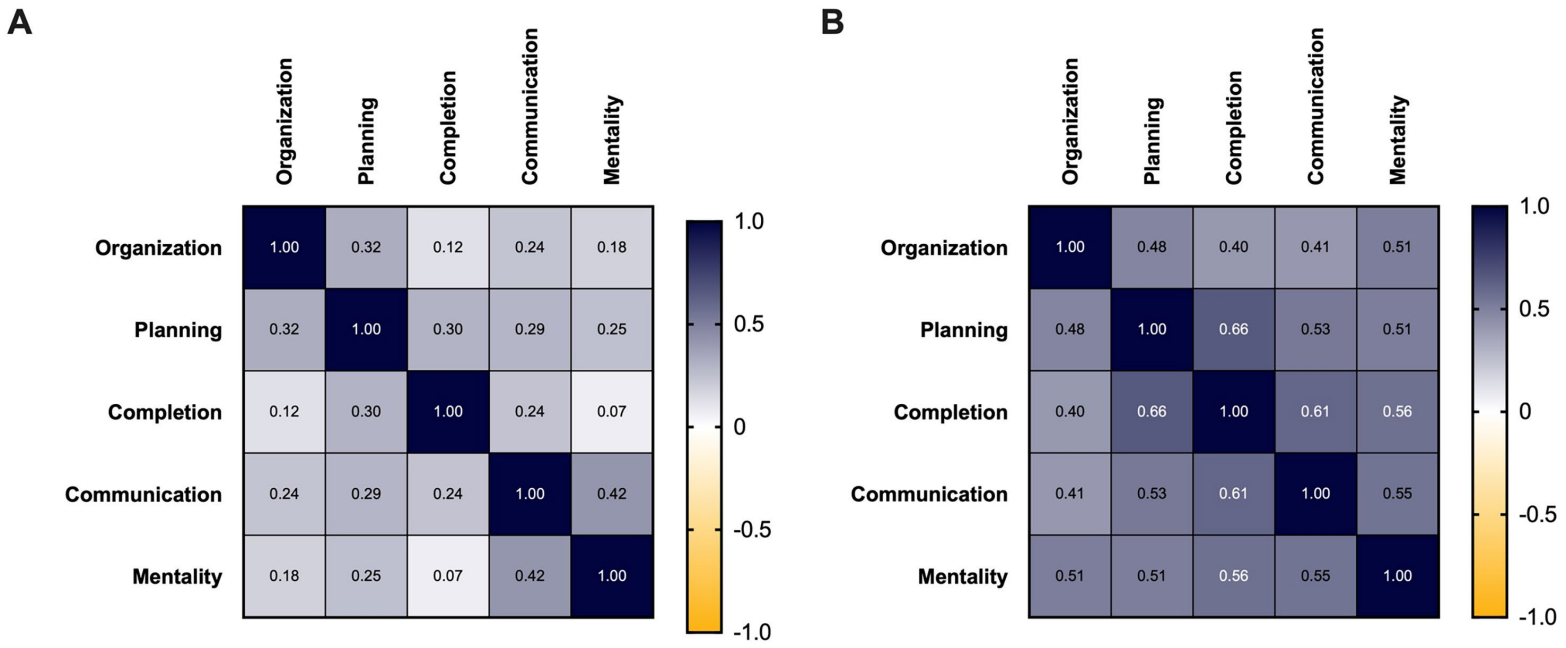
Correlations of skills measured in the EF survey. **(A)** Correlations between each skill assessed in the 6–12 EF survey demonstrated by Spearman’s *r*-value. Organization vs. completion *p* = 0.073, mentality vs. completion *p* = 0.329, all other comparisons *p* < 0.05. **(B)** Correlations between each skill assessed in the PS EF survey are demonstrated by Spearman’s *r*-value. All comparisons have *p* < 0.001.

Correlation analysis between subskills in the overall PS cohort revealed a significant correlation between all skill categories, indicating that all areas measured increase in concert (Figures 3A,B). When comparing skills in the 6–12 cohort all skills except completion with organization and mentality were significantly correlated (Figures 3A,B).

### 3.3 Age group effect

**Figure 4:**
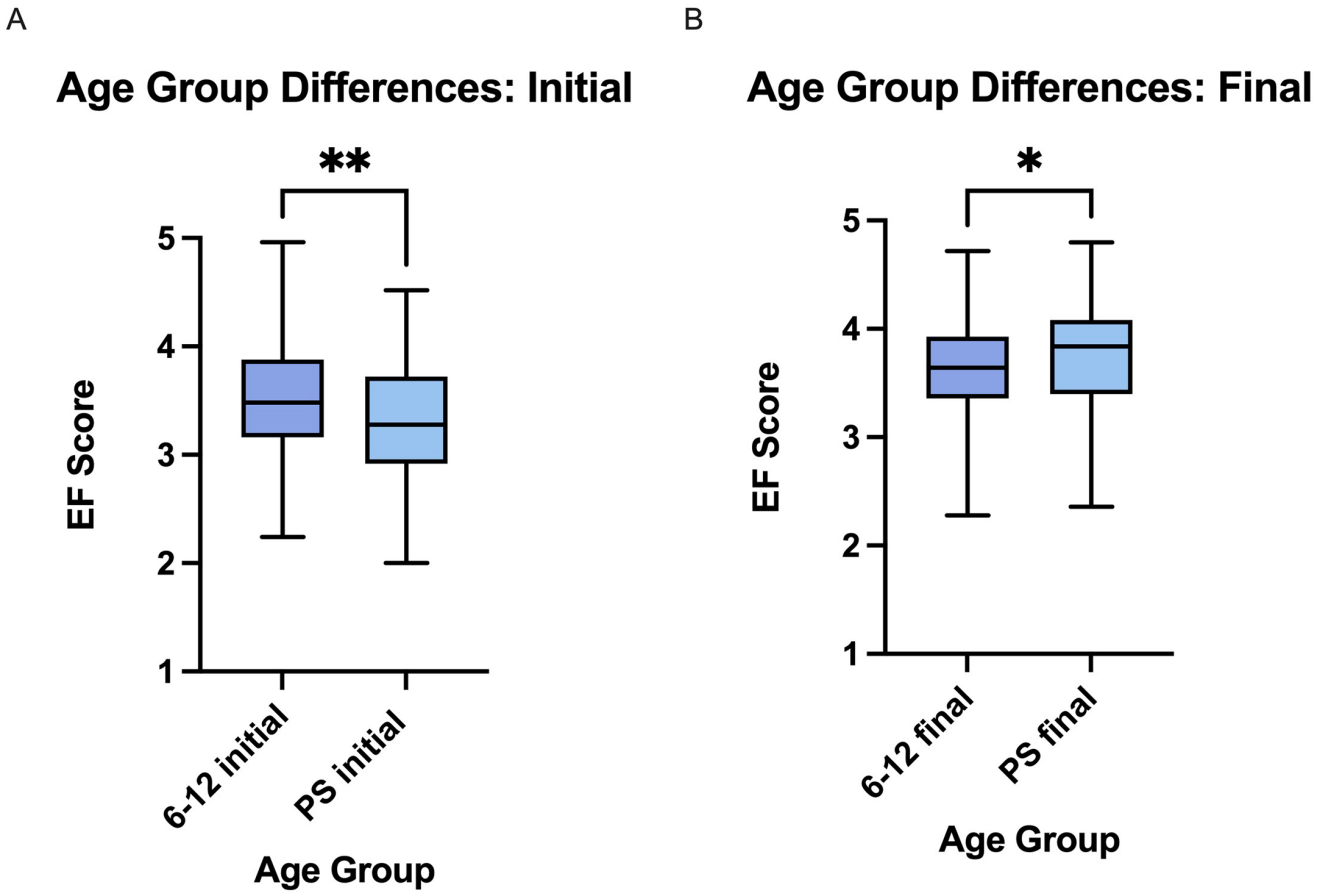
PS cohort had lower initial but higher final EF scores in comparison to the 6–12 cohort. **(A)** Upon comparison using an unpaired *t*-test it is apparent that the PS group had a lower initial score than the 6–12 group. 6–12 initial (*n* = 222) *M* = 3.50 (0.50), PS initial (*n* = 90) *M* = 3.30 (0.55), **p < 0.005. **(B)** The final PS score was higher than the final 6–12 score when compared by unpaired *t*-test. The center line of each box indicates the median, with bars reaching to the minimum and maximum values. 6–12 final (*n* = 222) *M* = 3.65 (0.44), PS final (*n* = 90) *M* = 3.76 (0.50), *p < 0.05.

The PS cohort experienced a greater increase in their reported EF skills in comparison to the 6–12 cohort (Figure 4). When combined across semesters, the average initial 6–12 EF score was higher (3.50 ± 0.50) in comparison with the PS (3.30 ± 0.55). Despite having a lower initial score the PS cohort consistently ended with a higher average final score than the 6–12 cohort (PS: 3.76 ± 0.50, 6–12: 3.65 ± 0.44) (Figure 4).

### 3.4 Sex effect

Initial EF scores did not differ significantly between sexes within the same age cohort (Figure 2). While both sexes had similar initial and final scores, there is still less significant growth seen in both the 6–12 and PS female cohorts when separated by semester and subskill (Tables 4, 5). The effect of sex may be influenced by the discrepancy in the number of females to males throughout all 3 semesters. When combined by age group and semester 28.84% of the population is female (Figure 1). This discrepancy is greater in the 6–12 group (26.13% female students) versus the PS group (35.56% female students) (Table 1).

## 4 Discussion

This study was a quality assessment project aiming to understand the impact of the UL program on EF skills in both an adolescent and college population (6–12 and PS). It was hypothesized that students enrolled in the program would report improvement in EF skills in both 6–12 and PS populations, with an emphasis on male improvement. Both age groups showed a significant increase in overall EF scores with the exception of the younger cohort in the S24 semester (Table 3). Despite reported overall EF improvement in both adolescent and college cohorts, there was a disparity upon stratification by sex, with males conveying greater EF gains than females. This aligns with the hypothesis that there would be a greater improvement in males, which may reflect the original design of the program for hyperactive males with ADHD (Table 3). Research demonstrates that movement-based programs can help students with EF skills gain greater working memory and inhibitory control; however, the longevity of these improvements may not be sustainable (Pontifex et al., 2013; Liu et al., 2020). Previous findings demonstrate that the increase in EF skills exists immediately following an acute bout of exercise (Liu et al., 2020). The current study suggests that combining EF skills training after a movement session may address this limitation, as improvements were observed across a semester (Table 3). Similar to Organizational Skills Training (OST) (Abikoff et al., 2013), Unstuck and on Target (UoT) (Kenworthy et al., 2014) and Homework, Organization and Planning Skills (HOPS) (Langberg et al., 2020) interventions, the students enrolled in the UL program demonstrated improvements in planning and organization (Tables 4, 5), suggesting that these domains may be responsive to skill-based training. The UL approach not only extends prior work in both the movement and skills-based domains, but also is one of the first studies to focus on both an adolescent and college population. This is an important aspect of the study as it allows the assessment of translatability throughout age groups.

### 4.1 6th–12th grade

Students in the 6–12 cohort largely reported improvements in their measured overall EF score (Table 3). However, stratification of the data indicates the trend is largely driven by the males (Table 4). Although males improved in overall EF growth across the S23 and F23 semesters, skill-level analysis revealed semester-specific patterns (S23 mentality, F23 organization, completion, and communication, S24 mentality; Table 4). Despite discrepancies during stratification, it is important to note that, with the exception of completion (S23, overall, female and male, S24 females), no group decreased in any semester or subskill; all showed trends upwards.

There were several factors that may have contributed to the variation seen in the 6–12 results. First, a substantial portion of the participants did not complete both initial and final surveys. Across both the 6–12 and PS cohorts combined, 61.62% (Figure 1) of students only completed one survey. It is important to note that this largely reflects survey non-completion rather than program attrition (Supplementary Figure 2; Supplementary Table 1). Contributing factors likely include competing end-of-semester academic demands, lower motivation to complete the assessment among younger participants, and the voluntary nature of survey administration within a non-clinical coaching context. This non-random missingness may limit the generalizability of the findings; however, this does not reduce the validity of the within-person results for students who completed both surveys.

The second factor that contributed to the noted variation is the substantially lower enrollment of female students. Within the S23, F23, and S24 semesters, there were 8, 35, and 15 females compared to 25, 72, and 67 males, respectively (Figure 1). With these sample sizes, the study was adequately powered to detect only very large effects in S23, small to medium effects in F23, and medium to large effects in S24. Overall, these power limitations may make null findings in females inconclusive, as true effects may be undetectable. However, the effect sizes in females tended to be smaller than in the males (Figures 3, 4), suggesting the pattern cannot be attributed to power limitations alone.

Lastly, there were marked differences in semester-to-semester subskill findings (Table 4), which suggests measurement instability. Several factors may contribute to this pattern, including variation in testing conditions, lack of question comprehension in younger students or lack of metacognitive skills to accurately answer the survey questions consistently. Survey administration was directed by company-wide guidelines; however, it was not strictly enforced as the assessment was designed for internal quality improvement. Variability in administration procedures may have contributed to inconsistent results. For instance, a younger student struggling with comprehension who was able to ask a clarifying question may have been able to complete the survey more accurately than one who did not fully understand what a question was asking. Coaches reported that younger students frequently struggled to understand certain survey questions, raising questions about the validity of the younger portion of this age group. It has been demonstrated in other studies of children and adolescents that language comprehension in self-report surveys is substantially lower in younger ages, particularly those under 13 years old (Eddy et al., 2011).

In particular, the category of “completion” had the lowest correlation with the other subskills in the 6–12 population (Figure 3), as well as the lowest overall internal consistency score (Supplementary Table 2). It was the only category with three reverse-coded questions; all the others had one or two. Considering that the Cronbach’s ⍺ scores substantially increased when reverse-coded scores were removed, it may be an indication that younger students in particular were producing erroneous responses to reverse-coded questions. Adolescent comprehension of reverse-coded questions is known to be unreliable and can contribute to measurement error (Antoniou and Alghamdi, 2024). The inconsistency in which skills showed improvement may be a reflection of reduced comprehension of questions rather than true intervention effects.

Furthermore, younger adolescents, especially with EFD, possess less developed metacognitive capacity and may not have the ability to accurately reflect on their growth (Weil et al., 2013). The disconnect between inconsistent student self-reports and largely positive parent feedback (collected anecdotally) may reflect the limitations of assessing this younger age group. In the future, integrating parent survey responses along with 6–12 students’ own responses would show a more comprehensive picture of the EF skill changes (Fisher et al., 2022).

Despite the measurement challenges detailed above, two interpretable patterns occurred. First, mentality (defined as increased confidence and reduced negative affect) increased across two semesters (Table 4). This pattern suggests that the questions pertaining to mentality could have been understandable to younger students, or that affective changes were more noticeable to 6–12 students than cognitive changes.

Second, groups with divergence in initial scores converge toward similar final scores (Figure 2 and Supplementary Figure 1). This convergence may be explained by improvements in metacognitive accuracy or ceiling effects inherent to the administration of Likert Scales, where adolescents tend to exhibit central tendency and end-avoidance, constraining the majority of scores to the 3–4 range (Raaijmakers et al., 2000). Together, these findings suggest that students enrolled in the 6–12 program express improvements in confidence and positive affect; however skill-specific assessment requires more developmentally and structurally appropriate assessments.

### 4.2 Post-secondary

Students in the PS program reported the most consistent increases in EF skill scores across all semesters and skill domains (Tables 3, 5). This stable pattern of score increases, in comparison to the younger cohort, may reflect several factors, including reality of consequences, greater metacognitive skills, and applicability of skills learned (Weiner et al., 2012; Weil et al., 2013; Zhuang et al., 2017). When students join the program as PS students, they often have experienced a real-life outcome of poor EF skills, such as failing a course, academic probation or paying to retake a course. These experiences may create a heightened awareness of EF deficits that increases their motivation to engage with the program. This is consistent with models of behavior change that emphasize the role of negative consequences in promoting adolescents’ motivation (Prochaska and DiClemente, 1983; Zhuang et al., 2017; Gaume et al., 2022). PS students are older and possess greater metacognitive abilities than younger students due to prefrontal cortex maturation throughout the early twenties (Weil et al., 2013). Greater metacognitive abilities may have contributed to more consistent self-report responses. This is consistent with the observation that PS students rated themselves lower at the initial assessment than the 6–12 students (Figure 4). Greater metacognitive abilities may have enabled more accurate self-assessment at both timepoints relative to the younger cohort.

Many of the PS students are living on their own with a greater degree of responsibility, including cleaning and cooking for themselves while attending classes. This allows for more opportunities to practice developing EF skills outside of the classroom. Daily planning tasks integrate multiple EF processes (Weiner et al., 2012) and frequent practice in routine tasks may be reflected in greater self-reported EF score increases. This is further emphasized by the increase in organization and planning subskills across semesters (Table 5), suggesting these frequently utilized skills may be the most pronounced in skills with greater opportunity for daily practice. Together, these factors suggest that PS students have a greater opportunity to practice and recognize EF skill application in daily life, which could be reflected in larger self-reported score changes. However, a critical limitation emerged; similar to the 6–12 cohort, there was significantly smaller EF score increases in PS females than males (Tables 3, 5). While the smaller number of PS females (*n* = 32) than males (*n* = 58) may limit the statistical power of this difference, the pattern cannot be ignored considering its consistency across age groups. The mechanism underlying reduced female growth may be attributed to an original male-centric program design, differential survey engagement patterns, or aspects of skill development not captured by the current assessment.

### 4.3 Sex differences

Analysis of both 6–12 and PS cohorts revealed males demonstrating larger score increases than females despite measurement noise. As a quality assessment study, identifying and understanding these sex based differences ensures equitable program design and development of appropriate outcome measurements for female participants. To understand the underlying contributing factors to sex-based differences in EF interventions, we examined literature on ADHD, as it is one of the most predominant EFDs. In most studies of ADHD, there is a larger proportion of males, which can be attributed to a lower incidence of diagnosis in females, and differences in symptom presentation that may reduce referrals for females to be tested or treated forADHD (Rucklidge, 2010; Skogli et al., 2013). As a result, most EF intervention programs, including UL, were initially designed around predominantly male samples and symptom presentations. This discrepancy between design and implementation populations may contribute to the differential patterns of score change observed between sexes Nevertheless, the results of this study indicate that females did demonstrate measurable score increases in specific EF domains, though potentially not in ways fully captured by the EF survey. When stratified by subskill, two semesters of PS females (Table 4). This suggests that score increases among PS females were most consistently observed in at least one critical EF domain. This pattern may reflect either a genuine differential response to the program in this domain, or that the survey was inadequate for detecting change in females in other categories.

Understanding patterns of female EF skill change is further complicated by the increased rate of sex-specific comorbidity that may affect accurate self-reporting (Young et al., 2020; Sokol et al., 2022). Females with EFDs, in particular ADHD, often have other internalizing comorbid mental health conditions, most notably anxiety (Young et al., 2020; Farhane-Medina et al., 2022). Critically, anxiety has been associated with negative self-evaluation bias, in which individuals may underestimate their own competencies on self-report measures, which may compress the observable range of score change (Sokol et al., 2022). This reporting bias suggests that smaller improvements in females may reflect, in part, differential self-report patterns rather than an absence of benefit from the program.

### 4.4 Limitations

This study should be interpreted as a quality improvement evaluation of a practice-embedded program rather than a controlled efficacy trial. The absence of a control group, randomization, and validated assessment tools remain major limitations of the study at hand. The findings here are intended to inform iterative program development and generate hypotheses for future research employing more rigorous and multi-informant experimental designs.

Statistically, there are many comparisons stratified by age group, sex, and semester. No correction for multiple comparisons was applied, given the exploratory nature of the analyses. As a result, some statistically significant subgroup findings may reflect type I error and should be interpreted as hypothesis-generating. Replication in adequately powered studies with confirmatory designs is warranted.

It should be noted that several of the authors are affiliated with UL and that the study was funded by the organization. While this is consistent within quality improvement research, where program employees are often involved in data collection and providing of contextual information, this affiliation introduces potential allegiance effects that may influence data interpretation. To mitigate this, all statistical analyses were conducted by an independent external contractor not employed by the organization using standardized methods. Furthermore, all findings, including null and negative results, are reported transparently. Nonetheless, findings should be interpreted with this context in mind.

This study had a few major limitations that limit the generalization of results and comparison to other studies. All data on student sex was reflective of their self- or parent-identified designation at enrollment and may not correspond to gender identity in all cases. Gender data was also not specifically collected. The use of a non-validated, internally developed assessment instrument limits comparability with other studies employing more established EF measures such as the BRIEF or BDEFS. The program’s cost-barrier limits access to the program and narrows the scope of enrollment to a predominantly middle-to-upper-class, Caucasian population, which reduces the generalizability of results. Furthermore, many students likely accessed concurrent services such as academic tutoring or therapy, which makes it difficult to attribute observed skill changes to the UL program alone.

## 5 Conclusion and implications

This study suggests that a personalized, movement-based approach is associated with increases in students’ reported EF skills in both adolescent and post-secondary populations. Students demonstrated significant increases in overall reported EF scores, with the most consistent score increases in the post-secondary student cohort. Males in both age groups demonstrated more uniform score increases; however, PS females had reported increases in the planning subskill, suggesting the female EF skill changes may not be fully captured by the current assessment tool.

The findings also indicate that a movement-oriented EF coaching program can feasibly be delivered, at scale, to two age groups with multiple access modalities. Coaches were able to engage with their students one-on-one, providing peer-support throughout the duration of the program. The goal of the program continued to be providing support in multiple EF domains, rather than one particular area, indicating that it is possible to develop sessions that integrate elements of different EF skills. Coaches were largely university students or recent graduates with varying levels of EF or clinical background. Despite often minimal background information, the training provided by UL in peer-support and EF skill development was sufficient to deliver the program across diverse caseloads. Furthermore, students and their families remained engaged in the program, with only 6.3% of students leaving the program mid-semester (Supplementary Table 1) in the three-semester period. The program has continued to scale since the study period with 406 students enrolled as of 2026, suggesting sustained demand and acceptability of the coaching model.

The data collection facilitated by this study indicated that it is feasible to administer a within-program survey in an EF coaching environment as long as it is well integrated into sessions. Frequent reminders to coaches, including emails and verbal reminders in their management meetings, allowed for greater participation. Given that the majority of students conducted their academic work digitally, the digital survey option yielded higher completion rates, and coaches reported greater ease of access. Aligning survey timing with academic calendars across multiple school districts and universities was critical to maximizing completion rates while avoiding overlap with exam preparation periods.

This study extends the current literature by suggesting that integrated movement and EF skills training may address the sustainability limitations observed in movement-only interventions. Furthermore, this is among the first studies to examine self-reported EF score changes following a coaching intervention across both adolescent and college-age populations. The core principles of this program: mentorship, movement and explicit skills training can be adapted for implementation in schools and community settings, expanding knowledge accessibility beyond fee-based programs. Following this study, UL has implemented female-centric offerings and multi-informant assessments as part of ongoing quality improvement efforts. Through the identification of programmatic strengths and areas for development, this study contributes to the broader effort to provide all students, regardless of sex, age, or socioeconomic status, with access to evidence-informed tools that will help them develop the executive function skills essential for academic and life success.

Future studies of program-integrated EF coaching should incorporate validated EF measures such as the BRIEF and BDEFS to align findings with current EF literature. To mitigate the impact of metacognitive limitations in younger populations and those with EFDs, multi-informant tools should be used to triangulate reports from coaches, parents, and students. Efforts should also be made to recruit larger female samples, as females remain underrepresented in EF research and insufficient sample sizes limit the ability to detect sex-specific patterns. Investigation of potential moderating or confounding factors such as stress and anxiety on self-report patterns may also help clarify the sex-specific differences seen in this study. Future research should consider designs that strengthen causal inference within the constraints of a program-delivery context. This could include incorporating mid-semester surveys to establish within-subjects baselines, comparing outcomes across varying lengths of program engagement, or comparing results with published normative EF data from validated assessment tools.

Overall, these findings provide a foundation for future research into movement-based EF coaching and highlight the importance of developing sex-specific programming and assessment tools for neurodiverse populations.

## Data Availability

The raw data supporting the conclusions of this article will be made available by the authors, without undue reservation.
