# Supplementary material for: The development and assessment of a movement-based coaching program on executive function skills: an exploratory quality improvement study
Source: Front Psychol. 2026 Mar 30;17:1761762. doi: 10.3389/fpsyg.2026.1761762 (PMC13071830; doi:10.3389/fpsyg.2026.1761762)
Supplement: Supplementary file 1 [file supplementary_file_1.docx]

Supplementary Material

# Supplemental Figure 1


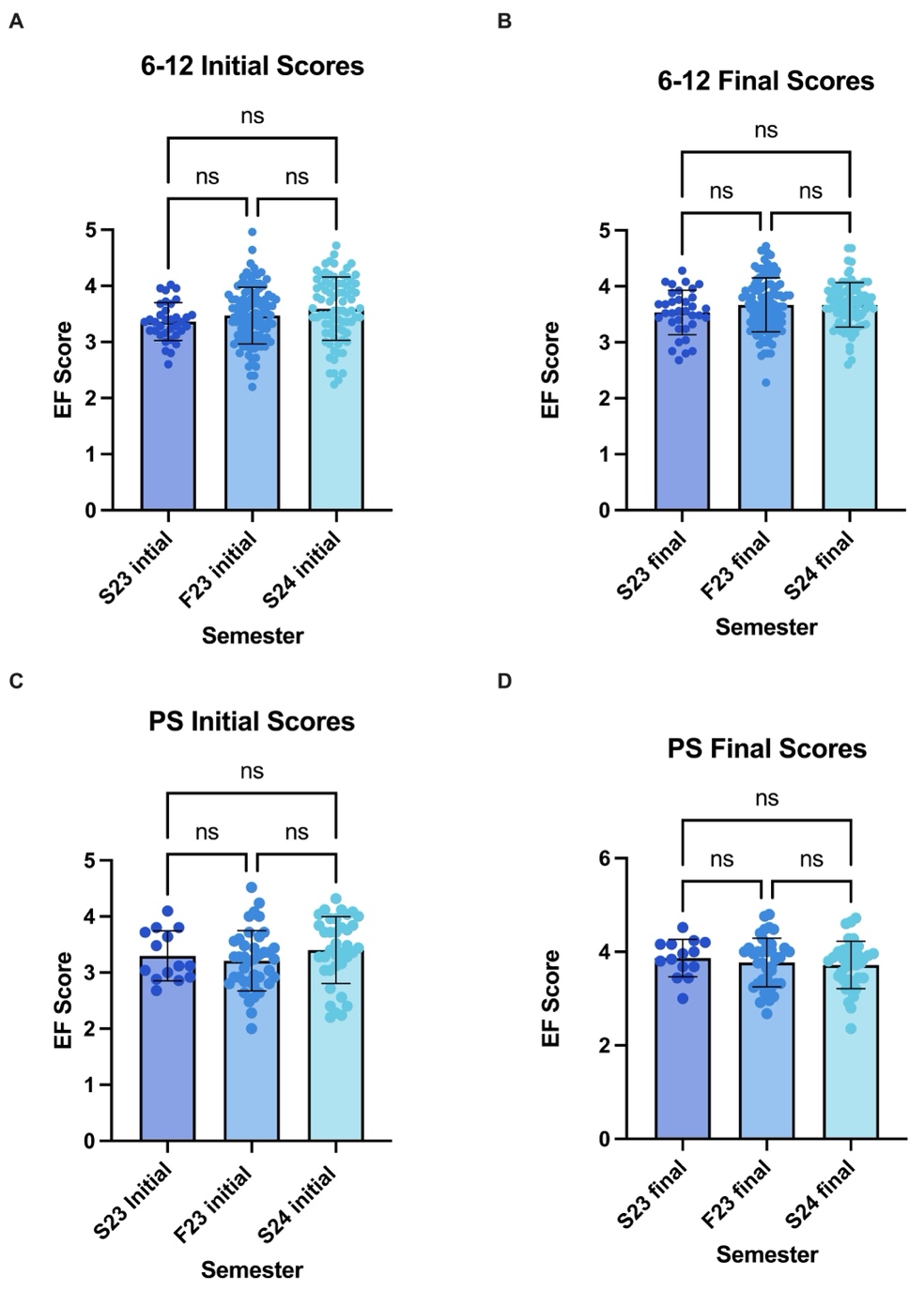


*Note.* **(A)** Comparison of initial scores within the 6-12 population between semesters. Analysis utilized a one-way ANOVA with a post hoc Tukey’s Test. Male and female data is combined for this assessment. Error bars represent the standard deviation from the mean. **(B)** Comparison of final scores within the 6-12 population, including male and female data between semesters. **(C)** Comparison of initial scores within the PS population, including male and female data between semesters. **(D)** Comparison of final scores within the PS population, including male and female data between semesters.

# Supplemental Figure 2


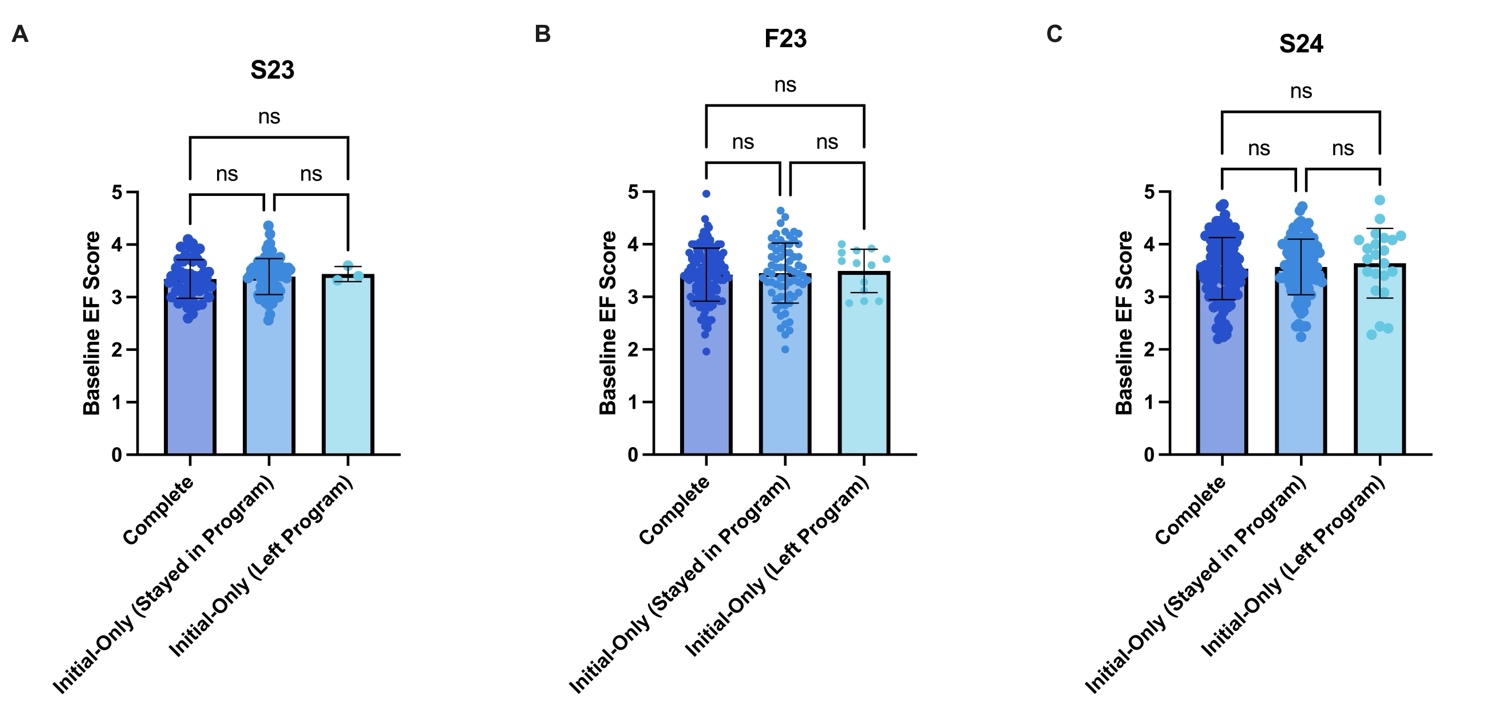


*Note.* **(A)** Comparison of initial EF scores between students in the Spring 2023 semester who completed both an initial and final survey, those who completed an initial survey but remained enrolled in the program and did not complete a final survey, and students who completed an initial survey and then left the program before completing a final survey. Analysis utilized a one-way ANOVA with a post hoc Tukey’s Test. Male, female, 6-12, and PS data are combined for this assessment. Error bars represent the standard deviation from the mean. **(B)** Comparison of initial EF scores in the Fall 2023 semester. **(C)** Comparison of initial EF scores in the Spring 2024 semester.

**3 Supplemental Table 1**

*Survey completion and attrition rates stratified by sex and age group*

|  | Spring 2023 | Fall 2023 | Spring 2024 |
| --- | --- | --- | --- |
| Overall (N = Initial survey completers) | 130 | 229 | 244 |
| Completed both | 47 (36.15%) | 146 (63.76%) | 119 (48.77%) |
| Initial only: stayed | 80 (61.5%) | 70 (30.57%) | 103 (42.21%) |
| Initial only: dropped | 3 (2.31%) | 13 (5.68%) | 22 (9.02%) |
|  |  |  |  |
| Male (n/N within gender) | 87 | 160 | 161 |
| Completed both | 37(42.53%) | 96 (60%) | 89 (55.28%) |
| Initial only: stayed | 48 (55.17%) | 53 (33.13%) | 56 (34.78%) |
| Initial only: dropped | 2 (2.3%) | 11 (6.88%) | 16 (9.94%) |
|  |  |  |  |
| Female (n/N within gender) | 43 | 69 | 83 |
| Completed both | 10 (23.26%) | 50 (72.46%) | 30 (36.14%) |
| Initial only: stayed | 32 (74.42%) | 17 (24.64%) | 47(56.63%) |
| Initial only: dropped | 1 (2.33%) | 2(2.9%) | 6 (7.23%) |
|  |  |  |  |
| Gender Comparison (3x2 test) |  |  |  |
| *p* | 0.0825 | 0.1869 | **0.0050** |
| *V* | 0.20 | 0.11 | 0.18 |
|  |  |  |  |
| 6-12 (n/N within age group) | 98 | 172 | 174 |
| Completed both | 33 (33.67%) | 107 (62.21%) | 82 (47.13%) |
| Initial only: stayed | 62 (63.27%) | 53 (30.81%) | 79 (45.4%) |
| Initial only: dropped | 3 (3.06%) | 12 (6.98%) | 13 (7.47%) |
|  |  |  |  |
| PS (n/N within age group) | 32 | 57 | 70 |
| Completed both | 14 (43.75%) | 39 (68.42%) | 37 (52.86%) |
| Initial only: stayed | 18 (56.25%) | 17 (29.82%) | 24 (34.29%) |
| Initial only: dropped | 0 (0%) | 1 (1.75%) | 9 (12.86%) |
|  |  |  |  |
| Age Group Comparison (3x2 test) |  |  |  |
| *p* | 0.4871 | 0.3407 | 0.1743 |
| *V* | 0.08 | 0.05 | 0.06 |

*Note*. For assessing proportional differences in completion between sub-populations within each semester, Fisher’s exact tests were conducted. Bolded text indicates p < 0.05. Cramer’s V values were calculated using the χ^2^ value, which was calculated separately from Fisher’s Exact test.

**4 Supplemental Table 2**

*Survey internal consistency measure for all questions and forward-coded questions*

| EF Subskill | ⍺ (all questions) | ⍺ (reverse-coded questions excluded) |
| --- | --- | --- |
| Organization | 0.64 | 0.82 |
| Planning | 0.53 | 0.73 |
| Completion | 0.15 | 0.57 |
| Communication | 0.67 | 0.88 |
| Mentality | 0.14 | 0.60 |

*Note*. Internal consistency assessment was calculated using Cronbach’s ⍺ for all questions and forward-coded questions, respectively. All semesters, age groups, and sexes are pooled.

**
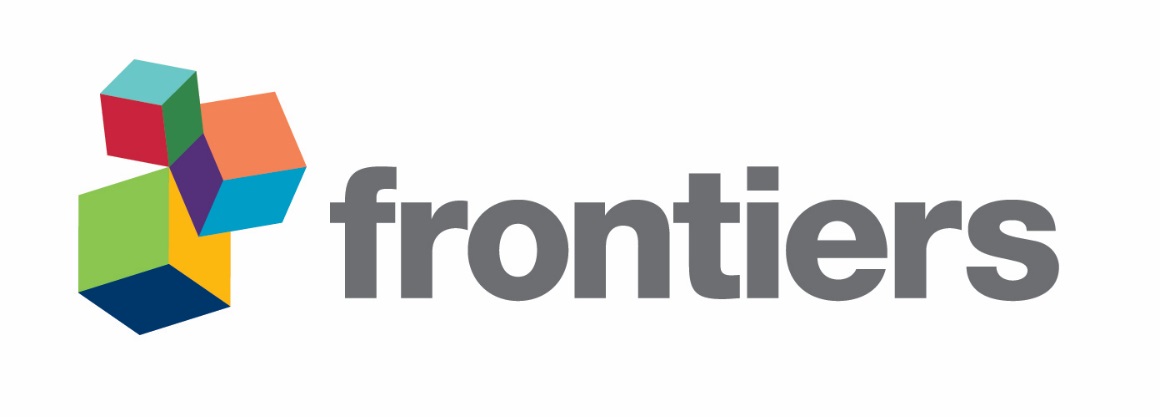
**
